# The fundamentals of eye tracking part 1: The link between theory and research question

**DOI:** 10.3758/s13428-024-02544-8

**Published:** 2024-12-12

**Authors:** Roy S. Hessels, Antje Nuthmann, Marcus Nyström, Richard Andersson, Diederick C. Niehorster, Ignace T. C. Hooge

**Affiliations:** 1 Experimental Psychology, Helmholtz Institute, Utrecht University, Heidelberglaan 1, 3584CS Utrecht, The Netherlands; 2 Institute of Psychology, Kiel University, Kiel, Germany; 3 Lund University Humanities Lab, Lund University, Lund, Sweden; 4 Tobii AB, Danderyd, Sweden; 5 Department of Psychology, Lund University, Lund, Sweden

**Keywords:** Eye movements, Gaze, Eye tracking, Theory

## Abstract

Eye tracking technology has become increasingly prevalent in scientific research, offering unique insights into oculomotor and cognitive processes. The present article explores the relationship between scientific theory, the research question, and the use of eye-tracking technology. It aims to guide readers in determining if eye tracking is suitable for their studies and how to formulate relevant research questions. Examples from research on oculomotor control, reading, scene perception, task execution, visual expertise, and instructional design are used to illustrate the connection between theory and eye-tracking data. These examples may serve as inspiration to researchers new to eye tracking. In summarizing the examples, three important considerations emerge: (1) whether the study focuses on describing eye movements or uses them as a proxy for e.g., perceptual, or cognitive processes, (2) the logical chain from theory to predictions, and (3) whether the study is of an observational or idea-testing nature. We provide a generic scheme and a set of specific questions that may help researchers formulate and explicate their research question using eye tracking.

This article is the first in a series on the fundamentals of eye tracking. The articles are aimed at individuals who are (one of) the first in their group, company, or research field to use eye tracking, with a focus on all the decisions one may make in the context of an eye-tracking study. Such individuals may come from academia (e.g., psychology, biology, medicine, educational science, computer science), commercial institutions (e.g., marketing research, usability, decision making) and non-commercial institutions (e.g., hospitals, air traffic control, military organizations). Note that this is not an exhaustive description of the target audience. More experienced eye-tracking researchers may find useful insights in the article series, or may find the article series a useful reference or hub to relevant research. One may either choose to start the series by reading the present article, but one may also skip to the other articles in the series first if they are of more immediate interest.

The study of eye movements and eye orientation has a long history, going back to the work of e.g., Ibn al-Haytham (Alhazen) and Aristotle (see Wade & Tatler, 2005; Wade, 2010; Płużyczka, 2018, for historical overviews). Why are eye movements of interest? Humans are animals with a fovea, meaning that only a small part of the visual field is represented at high resolution on the retina (see e.g., Tuten & Harmening, 2021). Acuity degrades with increasing distance from the fixation location (see e.g., Rosenholtz, 2016; Vater et al., 2022). Objects in the periphery are represented at a lower resolution compared to those in central vision. To perceive the visual world in detail, humans make saccades, typically multiple times per second, to project new areas onto the fovea. For individuals with normal vision, many activities, such as reading or searching for one’s favorite album in one’s record cabinet, cannot be accomplished without making saccades. However, this is not to say that peripheral vision is not important. A set of classic experiments reported by Graybiel et al. (1955) shows that activities such as figure skating, discus throwing, and slalom skiing are more impeded by blocking peripheral vision than by blocking central vision. Depending on the activity, central or peripheral vision may be more important (Rayner & Bertera, 1979; Nuthmann & Canas-Bajo, 2022).

The systematic recording and study of eye movements has been conducted since at least the early 1900s (Huey, 1900; Dodge & Cline, 1901; Dodge, 1903), and has tremendously progressed since^1^ (see e.g., Buswell, 1935; Ratliff, 1952; Yarbus, 1967; Merchant et al., 1974; Collewijn et al., 1975; Bour et al., 1984; Crane and Steele, 1985). At present, eye trackers are popular research tools in many research fields, including the study of the control of eye movements and its relation to vision (e.g., Kowler, 2011), reading (Rayner, 1998), visual search (Hulleman & Olivers, 2017; Godwin et al., 2021), scene viewing (Williams & Castelhano, 2019), working memory (Theeuwes et al., 2009), multimedia learning (van Gog & Scheiter, 2010), instructional design (Jarodzka et al., 2017), human interaction (Hessels, 2020), infant development (Gredebäck et al., 2009), autism (Falck-Ytter et al., 2013), visually guided behavior (Hayhoe & Ballard, 2005, 2014), human-computer interaction (Majaranta & Bulling, 2014), expertise (Gegenfurtner et al., 2011), etc.

As teachers in various eye-tracking courses and as supervisors to students (interested in) using eye trackers, we have found that the first question we ask after hearing one’s research question is often ‘do you need an eye tracker for your study?’ If an eye tracker is not necessary, we would advise not to include it in a study, as it may cost a lot of time, effort and resources, without delivering meaningful insights into a scientific problem. In research fields where eye tracking has been used for a long time, the role of the eye tracker is often evident. Yet, in research fields where eye tracking as a technique is relatively new, this may not be the case. Similarly, to individual researchers who are new to eye tracking, the role of the eye tracker need not be evident. In this article, we outline and give examples of what research questions can be adequately answered with the help of an eye tracker, focusing on the link between the goal of the study, the theory, and the eye-tracking data. The goal is for the reader to be able to answer the question of whether they need or can use an eye tracker for their study, or at least what they should consider to answer that question. It should be noted at the outset, however, that the question “do I need an eye tracker?” may seem deceptively simple, while the answer may be difficult to produce. Even if the answer is “yes, I need an eye tracker”, the precise insights that the eye tracker may yield may vary from study to study. And even if the answer is “no, I do not need an eye tracker per se”, the eye tracker may still yield insights in the context of other measurement techniques for one’s study. We see asking this question and producing an answer as an iterative process. The present article aims to serve as an aid in that process.

In what follows, we first discuss what the goal of eye-movement research may be, and how this goal may be achieved. Second, we give examples of eye-movement research from research fields with a strong relation between scientific theory and the eye-tracking data, and from research fields with a weaker theory–data relation. Based on these examples, we distill implications for formulating research questions that may be addressed with an eye tracker, or for deciding when to include an eye tracker in a study or not. The focus in this article is specifically on the research question. The next article in this article series will then focus on operationalizing research questions (Hooge et al., 2024b), approaching the relation between research questions and eye-tracking data from the empirical side. The articles hereafter will focus in more detail on setting up an eye-tracking study in the practical sense, including the choice for an eye tracker (Nyström et al., 2024) and tools for conducting a study with an eye tracker (Niehorster et al., 2024).

Throughout this article, various common terminology in eye-movement research will be adopted. The reader is referred to Table 1 for a refresher on these terms, or for references to more in-depth reading material. In particular, we recommend the reader unfamiliar with terms such as gaze, eye movement, saccade, or fixation to consult Hessels et al. (2018), who address definitions, and various scenarios in which these terms may be used differently. For operationalizations of various aspects of saccades, fixations, and so forth, we recommend readers to consult, e.g., Hooge et al. (2024b), section III in Holmqvist et al. (2011) or Bahill et al. (1975). For the present article, readers need not be concerned with understanding the exact meaning of eye movement measures in order to grasp the main points.

**Table 1:** Common eye-tracking terminology used in the present article

Terminology	Description
Eye tracker	Device used to estimate eye orientation relative to the head or gaze location in the world. Many different methods exist, including video-based eye trackers, electrooculography, and scleral search coils. See e.g., appendix B in Leigh and Zee (2015) and Chapter 2 in Holmqvist et al. (2011), as well as part 3 in this article series (Nyström et al., 2024).
Eye-tracking data	Anything observable by the use of an eye tracker, such as a gaze coordinate signal. The eye-tracking data may be used to estimate various gaze or eye-movement measures.
Gaze	May refer to the orientation of the eyes in the world (gaze direction) or location of the (binocular) fixation point in the world (gaze location, or point of regard). May also refer to movement of the eyes with respect to the world (e.g., gaze saccade, gaze shift). ‘Gaze behavior’ refers to the behavior of looking around in the world. See e.g., Hessels et al. (2018) and Hooge et al. (2024a).
Eye movement	Movement of the eye(s) with respect to the head. May include saccades, the vestibular-ocular reflex, the optokinetic reflex, fixational eye movements, smooth pursuit, etc. May be used as a container term, or with more specific meaning, with varied use in the literature (see e.g., section 2.5 in Holmqvist et al., 2011; Leigh and Zee, 2015; Lappi, 2016; Hessels et al., 2018).
Fixation	May refer to a period of no or little movement of the eyes with respect to the head or world. Often seen as the periods of interest for visual information processing. Definitions and operationalizations may depend on the research context (see Hessels et al., 2018).
Saccade	Fast eye movement to direct the fovea toward a new part of the visual world during which processing of new visual information is suppressed. Again, terminology may be strongly dependent on research context (see Hessels et al., 2018).
(Para)fovea	Regions of the retina that support central vision. The fovea is responsible for high-acuity vision at the fixation location. The parafovea circumscribes the fovea and supports slightly lower acuity vision further away from the fixation location (see e.g., Land & Nelson, 2012; Bringmann et al., 2018.
Periphery	The visual field beyond the parafovea (see e.g., Rosenholtz, 2016; Vater et al., 2022, for reviews). Note that different categorizations exist for the various regions of the visual field (Loschky et al., 2019).

## The why and how of eye-movement research

There may be many reasons for researchers to be interested in eye movements. As Leigh and Zee (2015) point out

“To the neurobiologist, the study of the control of eye movements provides a unique opportunity to understand the workings of the brain. To neurologists and ophthalmologists, abnormalities of ocular motility are frequently the clue to the localization of a disease process. Moreover, the visual and perceptual consequences of eye movements are important to both basic scientists and clinicians” (p. 3)

To this statement, we would add that to many (applied) researchers, eye movements may be seen as a proxy for varied aspects of cognitive functioning (e.g., memory, perception, problem-solving). An important first distinction that helps to understand the relation between scientific theory, research question, and eye-tracking data is between eye tracking for the observation and description of eye movements, and eye tracking for the probing of something else, e.g., perception, cognition, and so forth. An example study that focuses mainly on the description of eye movements is that by Dodge and Cline (1901), who report the angular velocity of eye movements. Other examples are Smeets and Hooge (2003), who investigate the variability in saccade amplitude and peak velocity and Van Renswoude et al. (2016), who investigate biases in saccade direction of infants and compare these results to data from adults. Eye movements, fixations, or gaze have been used as a proxy for comprehension during reading (Rayner et al., 2006), eye contact avoidance in socially anxious individuals (Wieser et al., 2009), attention guidance during learning (Jarodzka et al., 2013), multimodal speech perception (Yi et al., 2013), turn following and prediction in conversation (Casillas & Frank, 2017), and action prediction of infants at risk for autism (Braukmann et al., 2018).

A second important distinction is about the nature of the study on eye movements or gaze behavior. In general, research has been characterized as consisting of two modes, namely an idea-generating and an idea-testing mode. There are many other labels for this apparent dichotomy, including hypothesis-generating vs. hypothesis-testing, data-driven vs. hypothesis-driven, observational vs. experimental research, exploratory vs. confirmatory research, and night science vs. day science (e.g., Tukey, 1980; Jaeger & Halliday, 1998; Kell & Oliver, 2004; Concato, 2004; Yanai & Lercher, 2020). Thus, one could wonder whether a particular study is meant to generate (or observe) or test ideas (e.g., a hypothesis or theory). Examples of studies that mainly present observations are by Dodge (1903), who describes eye movements and categorizes them into five types; Klin et al. (2002), who describe gaze behavior to videos of social scenes of individuals with an autism diagnosis; Tatler (2007), who investigates the central fixation bias; Holleman et al. (2023), who describe gaze behavior during conversations between parents and children, and Hessels et al. (2020a), who describe gaze behavior during brief passing encounters in a hallway. Studies that explicitly test hypotheses or models are e.g., Hooge and Erkelens (1996), who test two models of the control of fixation duration during visual search; Kemner et al. (2008), who test two hypotheses for superior visual search performance in individuals with autism, and Moriuchi et al. (2017), who test two hypotheses for reduced gaze to the eyes in autism.

The dichotomy between idea-generating and idea-testing modes of science is a controversial description that has evoked strong opinions (see e.g., Platt, 1964; Meehl, 1978; Felin et al., 2021; Hessels & Hooge, 2021). Here we do not mean to uphold a dogmatic view of this dichotomy, but as will become apparent, it is a useful distinction when discussing the relation between research questions, scientific theories, and eye-tracking data. In addition to discussing the observation or idea-testing nature of eye-movement studies, we will also consider the ‘distance’ between a scientific theory and eye-movement or gaze measures. This distance can be considered in terms of the specificity of the predictions derived from the theory about eye movements, gaze behavior, or other aspects derived from the eye-tracking data. It can also be considered in terms of the number of assumptions that need to be made, or auxiliary theories to include (cf. Lakatos, 1978; Meehl, 1990), to make predictions at the level of eye-movement or gaze measures possible.

## Eye tracking research: from observation to stronger and weaker theory–data relations

In this section, we describe various research topics in which eye trackers have been prominent. The examples move from early observational research to research with a strong relation between theory and eye-tracking data (e.g., oculomotor control) to research with weaker theory–data relations or longer chains of reasoning from theory to eye-tracking data (e.g., instructional design). The described studies differ in the kind of predictions they make, and how these relate to the eye-tracking data: from predictions at the level of the eye tracker signal, to easily derived eye-movement measures, to overall statistics of gaze behavior. The examples are further characterized as being predominantly observational or of a strongly idea-testing nature, and on whether and which additional assumptions need to be made to go from theory to prediction. Based on these examples, and the principles that they illustrate, we will derive implications for formulating one’s research question, and assessing the relation between the scientific theory, research question, and eye-tracking data for one’s study.

The motivation for choosing these particular examples is the following. First, the empirical or the related theoretical work is well cited. Second, the examples come from prominent topics in eye-movement research. Thus, the studies mentioned in the context of these examples have been impactful in eye-movement research. Third, the examples are intimately familiar to the authors of the present article, which helps us best convey our message. Finally, the examples give a broad overview of the application of eye tracking, and are meant to cater to a diversity of interests among (new) eye-movement researchers. In our experience, many of the people we have met in the context of eye-tracking courses over the last years can identify their research with at least one of these examples (i.e., they are representative for many applications of eye tracking). We anticipate that to readers unfamiliar with these research topics, the examples may serve as inspiration. To readers more familiar with eye tracking, we expect that these examples may be familiar, or may spark new insights even after years of conducting eye-movement research.

### Example 1: Early observational studies of eye movements

The first example illustrates the importance of observational studies in eye movement research. Many studies that focus on eye movements per se are of an observational nature. That does not mean, however, that the description of eye movement behavior is the sole goal behind such studies. Two examples from the early 1900s make this point clear. Huey (1900) conducted a study on eye movements during reading and describes what we might now call the saccade-fixation-saccade sequence, amplitude of saccades, fixation duration, and the location of fixations with respect to the lines of text. Huey notes explicitly that he “... planned to make an analysis and description of the reading process” (p. 283). However, the reason why he delivered this description was more fundamental. As he writes: “To explain fully the ‘how’ of reading would be to write a treatise on the senses and intellect, and, in fact, to say the last word on many of the fundamental problems in psychology. The present study is but a beginning of what should be done in this field. It is hoped that it may at least make the general subject easier of approach” (p. 284). That is, Huey saw the description of eye movements during reading as a necessary step to understanding the reading process, and perhaps human cognition in general.

Similarly, Dodge (1903) described and classified five types of eye movements in his study. He writes that “The limitations of available apparatus have necessarily restricted the scope of the investigation; but it seemed to me that enough of general physiological interest had been obtained to warrant its publication as a contribution to the classification of the eye movements” (p. 307). Yet, the reason why Dodge took to describing eye movements in such detail was that it was of theoretical importance to the visual perception of space (see also Dodge, 1900). Thus, while the studies by Huey and Dodge were observational and descriptive in nature, they bore on larger problems of theoretical interest to them. Observation and description of the eye movements were necessary first steps.

### Example 2: Oculomotor control

The second example illustrates a research field where there is a strong theory-data relation. Different models are proposed that make predictions at the level of the eye-tracking signal or for a singular aspect of gaze behavior, which can be directly compared in empirical tests.

The early observational studies of eye movements by Dodge and Cline (1901) and Dodge (1903) lead us naturally to the study of eye movements to gain insights into oculomotor control and the modeling of the oculomotor control system. Studies in this area of research have focused on the capability of the oculomotor system, e.g., in terms of tracking targets in the world, the neuroanatomy and neurophysiology of the oculomotor system, as well as formally modeling the oculomotor control system (see e.g., Robinson, 1968; Leigh & Zee, 2015; Robinson, 2022, for reviews).

A good example is the study by Robinson (1973), who presents four models for the saccadic eye movement control system. These models are increasingly more complex to account for experimental results, i.e., eye-tracking recordings of human saccadic eye movements following a fixation target jumping between different locations. The development of the models is done taking into account neuro-anatomical structures and neurophysiology. The models predict saccadic eye movements in response to a defined target movement. What is important to realize is that these predictions are at the level of the eye tracking signal, and can be directly tested with experimental studies to corroborate, falsify, or modify the models. After discussing the utility of the first three models, Robinson (1973) introduces a fourth model, which is conceptual and not empirically tested. In light of this fourth model, more complicated eye movement phenomena are discussed and theoretical considerations of earlier models are evaluated.

Studies on oculomotor control may also focus on temporal aspects of gaze behavior (Vaughan, 1982; Hooge & Erkelens, 1996, 1998). For example, Hooge and Erkelens (1996) investigated how the duration of a fixation is controlled during visual search, specifically the search for a circle among letter C’s. The authors considered two models, namely the process-monitoring model (Rayner, 1978) and the preprogramming model (Vaughan, 1982). The process-monitoring model holds that a saccade is planned only after the foveal target (i.e., the fixated item) is processed, thus controlling fixation duration directly. The preprogramming models hold that the fixation duration is preprogrammed and independent of the analysis of the foveal target. Hooge and Erkelens (1996) find that on 5–55% of the trials, the target was fixated, a saccade was made to a next item, and the target was then refixated. The analysis of the foveal target seems not to have been completed upon initiating the saccade away from the target, thus falsifying the process-monitoring model. Hooge and Erkelens (1996) moreover consider their results in the light of two variants of a preprogramming model, and a mixed-control model including both a preprogramming and a process-monitoring component. They suggest that “control of fixation duration in a simple search task ... is indirect. Adjustment of fixation duration is based on the expected difficulty ... estimated during previous fixation” (p. 976).

In the example by Hooge and Erkelens (1996), the various models make competing predictions about a specific aspect of gaze behavior, namely the durations of fixations under various conditions. As fixation duration is easily derived from the eye-tracking signal (i.e., the distance between theory and eye-tracking data is small), the models can be directly tested against each other in a straightforward manner.

Since the study by Hooge and Erkelens (1996), a substantial body of empirical research has focused on investigating the precise mechanisms controlling fixation duration across a variety of visual-cognitive tasks, including variants of the C-search task (e.g., Trukenbrod & Engbert, 2012), scene perception (e.g., Einhäuser et al., 2020; Henderson & Pierce, 2008), and sentence reading (e.g., Dambacher et al., 2013), with the development of computational models being an integral part (e.g., Trukenbrod & Engbert, 2014; Walshe & Nuthmann, 2021).

### Example 3: Reading

In the following example from reading research, we illustrate (1) the role of generic assumptions about eye movements and gaze in linking scientific theory to research question and (2) the role of computational modeling in testing predictions derived from theory. Reading can be approached from at least three perspectives, shaping how eye movements are studied and analyzed: the first emphasizes perception and motor control, the second is rooted in cognitive psychology and views reading as a complex information acquisition process, and the third focuses on using eye-movement measures to test psycholinguistic hypotheses about written language processing (Radach & Kennedy, 2004). Our example illustrates the cognitive perspective, through which gaze behavior and eye movements in reading have been extensively studied for decades (Hyönä & Kaakinen, 2019; Rayner, 1978, 1998).

An ongoing theoretical controversy revolves around whether words are processed one by one in a serial manner (Reichle et al., 2009) or if multiple words can be processed in parallel (Snell & Grainger, 2019). This controversy arises from differing assumptions made by computational models of eye-movement control in reading. In contrast to the strictly serial E-Z Reader model (Reichle et al., 1998, 2003), models like the SWIFT model (Engbert et al., 2005; Schad & Engbert, 2012) and the more recent OB1-Reader model (Snell et al., 2018) assume that words within the perceptual span (McConkie & Rayner, 1976) are processed in parallel.

Empirical tests of serial versus parallel word processing are linked to two classic assumptions proposed by Just and Carpenter (1980) regarding the relationship between eye movements and cognitive processing. According to the eye–mind assumption, “the eye remains fixated on a word as long as the word is being processed” (p. 330). This assumption is accompanied by the immediacy-of-processing assumption, which suggests that there is “no appreciable lag between what is being fixated and what is being processed” (p. 331).

Word length, frequency, and predictability of the currently fixated word have reliable effects on the time spent on this word (see Clifton et al., 2016, for a review). Finding such immediacy effects of “The Big Three” variables of lexical processing (Kliegl et al., 2006) on the current fixation duration lends clear support for the immediacy and eye-mind assumptions. Notably, in subsequent work, Carpenter and Just (1983) relaxed the assumptions somewhat by allowing influences from previously fixated words. Indeed, research has shown that the processing of the preceding word can ‘spill over’ onto the current word, increasing its fixation time (e.g., Kliegl et al., 2006; Rayner & Duffy, 1986; White, 2008), though note that spillover effects do not always occur (e.g., Carpenter & Just, 1983.

Considering that both present and past words can influence measures of fixation duration in reading, the question arises whether future words can exert a similar influence. Parafoveal-on-foveal (PoF) effects specifically address how the next word(s), typically situated in the parafovea, may influence the fixation time on the current word (i.e., the word in the fovea). Evidence supporting PoF effects would challenge the immediacy-of-processing and eye-mind assumptions. Moreover, while PoF effects are compatible with parallel-processing models like SWIFT, they are incompatible with the architecture of serial-processing models like E-Z Reader. Whether lexical and semantic PoF effects exist and whether they represent genuine phenomena or artifacts has been a matter of fierce debate (Brothers et al., 2017; Kennedy, 2008; Kliegl et al., 2006; Murray et al., 2013; Reichle & Drieghe, 2015).

In these examples from reading research, as in the previous example on oculomotor control, there is a strong link between the theory, computational models, and the eye-movement or gaze measures. The predictions delivered by the theory and/or computational model link eye-movement or gaze measures, easily derived from the eye-tracking data, directly to characteristics of the stimulus material (i.e., the words in the reading material). The eye-tracking data may be used in a straightforward manner to test, falsify, or modify the theory and/or computational model. Specifically, classic assumptions in eye-movement research (eye-mind assumption and immediacy-of-processing assumption) are tested and falsified or nuanced in the context of eye movements and gaze behavior during reading.

### Example 4: Scene perception

In experiments on scene perception, the stimuli range from line drawings and computer-generated images to photographs and videos of real-world scenes. A great deal of research has investigated the factors that control *where* and *how long* people look in scenes (Kümmerer & Bethge, 2023; Nuthmann, 2017; Williams & Castelhano, 2019). In the context of this research, fixation durations may provide temporal estimates of cognitive processing times, while fixation locations may approximate the locus of cognitive processing and attention, albeit not under all circumstances (see Irwin, 2004, for a critical discussion).

The following example illustrates how an eye tracker may be used to test phenomena from an applied problem, namely the reliability of eyewitness testimonies. Moreover, it shows how eye tracking may be used to test straightforward hypotheses about observable phenomena, not just predictions based on full-fledged theories or computational models as in the reading example above.

One applied research question on scene perception, originating from eyewitness research, concerns a phenomenon known as the ‘weapon focus effect’ (WFE) (Loftus et al., 1987). The WFE describes how the presence of a weapon can compromise observers’ memory for the appearance of the individual holding the weapon (see Fawcett et al., 2013, for a meta-analysis). Loftus et al. (1987) proposed two hypotheses to explain the WFE. According to the arousal/threat hypothesis, the WFE occurs due to the threat posed by the weapon. In contrast, the unusual-item hypothesis suggests that weapons are unusual in most contexts, akin to the presence of an octopus in a farmyard (Loftus & Mackworth, 1978). Both explanations share the idea that observers’ attention shifts from the perpetrator to the weapon itself, hence the term ‘weapon focus.’ In much of the research on the memory component of the WFE, this attention shift is inferred from differences in memory accuracy (e.g., Harvey & Sekulla, 2021; Pickel, 2009) or assessed through self-report (Erickson et al., 2014). Yet, only a few studies have directly examined the postulated attentional shift using eye tracking. Notable exceptions include the classic study by Loftus et al. (1987) and, more recently, a series of experiments by Körner et al. (2023, 2024).

Using slide shows, Loftus et al. (1987) found more and longer fixations on the weapon compared to a neutral object, although no data was reported for looks to the perpetrator. Körner et al. (2023) adapted videos from an existing study (Pickel & Sneyd, 2018) in which the presence of a weapon was associated with a clear reduction in memory performance. For one of their experiments, Körner et al. (2023) converted the videos to slide shows to mimic the methodology used by Loftus et al. (1987). Replicating this study, observers spent more time looking at the weapon than the neutral object. However, this increase in total viewing time to the weapon did not come at the cost of viewing time to the perpetrator. Importantly, when videos were used instead of slide shows, there was no evidence supporting the postulated attention shift. These differences in results indicate that it is important to study the attentional effects of weapons under more representative viewing conditions (i.e., dynamic scenes). In addition, Körner et al. (2023) found that self-reported relative total viewing times for the critical object and the people in the scenes were not very accurate representations of subjects’ actual gaze behavior. Their time-course analysis on the gaze data from the experiment with videos revealed that both the weapon and the neutral object drew gaze primarily at the beginning of the scene as the perpetrator entered the room, but were not looked at much thereafter. Contrary to predictions, the presence of a weapon did not lead participants to remember fewer details about the perpetrator’s appearance in either experiment (see also Körner et al., 2024).

The ‘weapon focus effect’ example illustrates that there does not need to be an extensive theory linking the phenomenon – compromise of an observer’s memory for an individual holding a weapon – to gaze behavior. The two hypotheses put forward by Loftus et al. (1987) both suggest the same mechanism of action, involving an attention shift from the perpetrator to the weapon, which is expected to manifest in gaze behavior. Eye tracking provides an objective way to test this hypothesis, as opposed to e.g., self-report questionnaires.

As a final note, the study by Körner et al. (2023) also attests to the importance of the representativeness of the viewing conditions for the problem at hand (see also Holleman et al., 2020). While this is not the general topic of the present article, it is at the heart of the following example on visually guided task execution.

### Example 5: Visually guided task execution

The present example serves to make a number of points. One is that descriptive studies are important also for research topics with a potentially large distance between theory and gaze behavior (e.g., as compared with the example on oculomotor control above). Moreover, the present examples illustrates the scope and breadth of predictions about gaze behavior that can be made on the basis of the theory.

As Hayhoe (2017) points out, an “integrative view of vision in its behavioral context” requires considering vision and action simultaneously. A substantial amount of eye-movement studies have been conducted on the topic of gaze behavior during the execution of visually guided tasks. One finds this research topic under various terminology, including e.g., visually guided actions (Hayhoe, 2017), visual routines (Ullman, 1996; Hayhoe, 2000), eye movements in natural behavior (Hayhoe & Ballard, 2005), task-control of eye movements (Hayhoe & Ballard, 2014), task-related gaze behavior (Hessels et al., 2023), and gaze–action coupling (Hessels et al., 2024). While a lot of eye-movement research may be characterized as ‘task-related’, we are concerned specifically with wearable eye-tracking studies during the execution of activities of daily life (see Land & Hayhoe, 2001, for a good starting point). Using example studies from this research topic, we illustrate (1) the importance of descriptive studies, (2) the scope of application of a theoretical framework, and (3) less direct predictions about gaze behavior than in the previous examples on reading and scene perception.

In pioneering studies on visually guided task execution, subjects were equipped with mobile eye-tracking gear while they drove a car (Land & Lee, 1994), copied a block model (Ballard et al., 1995), prepared tea (Land et al., 1999), washed their hands (Pelz & Canosa, 2001), or made a sandwich (Hayhoe et al., 2003). These studies revealed that participants’ gaze was closely timed to the manual actions they were performing. For example, prior to grabbing a tea cup, the handle would be fixated, while the faucet would be fixated prior to pouring water in a kettle. Land et al. (1999) and Pelz and Canosa (2001) concluded that most fixations were on locations in the world immediately relevant to the task, with some fixations related to upcoming actions. In recent years, the study of visually guided task execution has been extended to, e.g., foot control in rough terrain (Matthis et al., 2018), crowd navigation (Hessels et al., 2020b), assembling a camping tent (Sullivan et al., 2021) or stair walking (Ghiani et al., 2023).

The early descriptive studies by Land, Pelz, Hayhoe, and colleagues have led to a theoretical framework of task-related gaze behavior summarized in e.g., Hayhoe and Ballard (2005) and Hayhoe and Ballard (2014). The basic principles are that “fixations are tightly linked in time to the evolution of the task” and that “highly task-specific information is extracted in different fixations” (Hayhoe & Ballard, 2005, p.189). While “the timing and choice of gaze targets [...] are intimately linked with ongoing behavior”, “modeling of the deployment of these fixations has been very difficult because they depend on characterizing the underlying task structure” (Hayhoe & Ballard, 2014, p.R622). This problem is simplified by assuming that complex tasks can be broken down into simpler, independent sub-tasks. For example, the task of making tea can be understood as a series of manual actions (grabbing a cup, pouring water into a kettle, etc.) that each must be completed in turn, and have a corresponding relevant fixation location in the world.

What does the theoretical framework predict? First of all, if one has a model of the task being carried out (i.e., the required manual actions and their order), one can predict where a person will look at each point in time when carrying out that task. For example, Hayhoe and Ballard (2014, p.R626) explain that “Sandwich making has much underlying regularity to its observed behavior, and it’s possible to infer the underlying task structure very accurately by incorporating the observable data, such as the gaze location, hand position, hand orientation, and image features as well as the prior sequence of states of the task”. Thus, in certain tasks, such as sandwich making, the task structure may be evident, which allows accurate prediction of the spatiotemporal gaze behavior in terms of fixation locations in the world related to task execution. Note that Hayhoe and Ballard (2014) acknowledge that there may be some arbitrariness in inferring a task structure, and that ideally, a more formal theory of task structure would be desirable. Moreover, the assumption of independent sub-tasks is likely an oversimplification: “For the most part, a new visual computation will involve a shift in gaze. This is not always true, for example, when spatially global visual information is needed, or when peripheral acuity is good enough to provide the necessary information without a gaze shift” (Hayhoe & Ballard, 2014, p.R623). In sum, for tasks for which a task structure is evident or for which formal models exist, *and* where sub-tasks have different relevant fixation locations in the world, one can predict gaze behavior. However, not all activities that humans encounter on a daily basis may fit this scheme (see e.g., Ghiani et al., 2024).

There may also be many situations where humans execute multiple tasks simultaneously, or at least interleaved. Hayhoe and Ballard (2014) further provide an account how the perceptual arbitration process unfolds in such scenarios, i.e., which ‘task module’ is updated by fixating a relevant location in the world. The central principle for the arbitration process is that “gaze deployment depends on both reward and uncertainty” (p. R624). Vision is seen as a serial process where new information can only be acquired for one task module at a time. The other task modules rely on potentially noisy estimates from memory. “The gaze location chosen is the one that reduces [the] reward-weighted uncertainty the most” (p. R624). Thus, the relative reward for successfully completing each task, as well as the uncertainty of the state of the world relevant to that task predict whether gaze is likely to be allocated in service of that task. While this account may not perfectly predict individual gaze shifts, it does predict changes in the statistics of gaze behavior as a function of uncertainty and relative reward associated with different tasks. Several studies have tested such hypotheses empirically (see e.g., Sullivan et al., 2012; Tong et al., 2017).

The research on task-related gaze behavior outlined above makes a number of points clear. First, descriptive studies are tantamount to understanding and modeling task-related gaze behavior. Second, the theoretical account linking task execution to gaze behavior only holds for tasks with a clear, inferable task structure. It is unclear how much of daily activity fits this category. In other words, it is unclear how widely applicable the theory is. Third, for concurrent execution of tasks, the theoretical account does not predict individual gaze shifts (at least not without making additional assumptions), but rather statistical aspects of the aggregate gaze behavior, where uncertainty and reward are important determinants. Here, the link between theory and eye-tracking data is less direct than for the examples from reading and oculomotor control above.

### Example 6: Expertise

We move on to a slightly vaguer concept, namely that of expertise. This section first illustrates a specific application of eye tracking to understand expert performance in chess and the assumptions that need to be made to interpret the eye-tracking data in a meaningful manner. Secondly, the section illustrates the role of eye tracking in the formulation and testing of domain-generic theories of expertise. This example illustrates how eye tracking may be used in the context of a potentially ‘vague’ theoretical concept such as expertise.

Simon and Chase (1973) have argued that “As genetics needs its model organisms, ..., so psychology needs standard task environments” and according to them, chess is an excellent model environment for studying e.g., expert performance. In 1946, de Groot (2008)^2^ pioneered the study of thought and choice processes in chess. Subsequent work by e.g., Simon and Chase (1973) led to the idea that the advantage of an expert chess player results from “quickly perceiving chess positions”, “reconstructing them from memory”, and that “the patterns that masters perceive will suggest good moves to them” (p. 402–403). According to them, the key to understanding chess skill is to understanding the perceptual processes of the expert chess player. Reingold et al. (2001) used eye tracking “to provide direct evidence for the hypothesis that a perceptual advantage is a fundamental component of chess skill”.

In order to test the hypothesis of a chess-specific perceptual advantage for expert chess players, Reingold et al. (2001) conducted a number of experiments. They first predicted that the expert’s perceptual advantage should be evident from a larger visual span specifically for chess configurations. To test this, they used a change detection task by Rensink et al. (1997) and a gaze-contingent window technique used by, e.g., Reder (1973) and McConkie and Rayner (1975) in the context of reading research. The task was to detect which chess piece was changed between two alternating presentations of the chessboard configuration. The gaze-contingent window allowed the researchers to replace all chess pieces by gray blobs when they were more than a specified distance away from the gaze position on screen. These methods combined, the authors were able to estimate the visual span for chess-related and chess-unrelated (i.e., random) configurations on the chessboard. The visual span was operationalized as the number of squares from which the participants could effectively detect which chess piece was changing between flashes of the chessboard. Unlike novice or intermediate chess players, expert chess players had a much larger visual span for chess-related but not chess-unrelated configurations of the chess board. That is, experts could perceive much larger chess-related configurations in one go than non-experts.

Second, Reingold et al. (2001) derived predictions about gaze behavior from the work of e.g., Simon and Chase (1973) and the principle of chunking proposed by Miller (1956). If experts have internalized familiar or common configurations of multiple chess pieces into single units (‘chunks’), this may lead to faster recognition of the chessboard configuration. Reingold et al. (2001) reasoned that if experts encode the chessboard configuration in chunks rather than by individual pieces, this should result in fewer fixations on individual chess pieces and more fixations between chess pieces. To test this, they conducted a check-detection task, i.e., determining whether the king chess piece is under attack by another chess piece. Here, experts were faster overall, and made fewer fixations on individual chess pieces and the chess configuration overall than novice or intermediate players. Reingold et al. (2001) concluded that their findings are “strong evidence for a perceptual encoding advantage for experts attributable to chess experience, rather than to a general perceptual or memory superiority” (p. 48).

In the study by Reingold et al. (2001), the eye tracker was used in two different ways. First, the eye tracker was used as a tool to implement gaze-contingent changes to the display. Second, the eye tracker was used to test predictions about gaze behavior derived from previous research on expertise in chess. The necessary principles or assumptions needed to link expertise to gaze behavior were derived from (1) the chunking principle by Miller (1956), (2) the modeling work by Simon and Chase (1973) relating perceptual processes to phenomena of expertise in chess and (3) the assumption that gaze location reveals some information about what constitutes a chunk to the observer (i.e., an individual chess piece or a configuration). The predictions were about differences in aggregate measures of gaze behavior between groups.

Besides the study by Reingold et al. (2001), a substantial amount of eye-movement research has been conducted on visual expertise (see e.g., Gegenfurtner et al., 2011; Brams et al., 2019, for reviews). Gegenfurtner et al. (2011) conducted a meta-analysis in this field and related the eye-tracking findings to three different theories proposed to account for visual expertise (see their Table 1), namely (1) the theory of long-term working memory, (2) the information-reduction hypothesis and (3) the holistic model of image perception^3^. The three theories make different predictions about differences in gaze behavior between experts and non-experts (see their Table 8). Gegenfurtner et al. (2011) report that across studies, substantial differences in gaze behavior between experts and non-experts are observed, for example with regard to fixation durations (shorter for experts) or the number of fixations (more fixations on task-relevant areas and fewer fixations on task-redundant areas for experts). All three theories find at least some predictions supported by empirical research, although the visualization type, task complexity and domain of expertise seem to be important moderators of differences in gaze behavior between experts and non-experts. The latter point is corroborated by Brams et al. (2019), who conclude that:

“... all three theories or some combinations of these theories may explain some aspects of expert performance, depending on the specific task. The field is, therefore, in strong need of a more integrative theory, which encompasses the basic building blocks highlighted by each theory but with sufficient empirical support” (p. 43)

Thus, it seems that eye-movement research is essential to testing, developing, fine-tuning theories on expertise in general, or for domain-specific applications. It seems unlikely that a domain-generic theory may be formulated that predicts gaze behavior of experts and non-experts across most domain-specific contexts.

### Example 7: Instructional design

The final example is from the domain of instructional design in educational science. In this example, the distance between the scientific theory and the predictions that can be evaluated using eye-tracking data is substantial. The theory does not explicitly make predictions about eye movements or gaze behavior. Through additional assumptions, some of which are contested in other research fields, the use of eye tracking is motivated.

In instructional design, a prominent theory is the cognitive theory of multimedia learning (CTMML; Mayer, 2021). As Jarodzka et al. (2017) outline, CTMML is one of most important theories in instructional design and eye-tracking can be used as a tool for testing such learning theories in educational practice (see also van Gog & Scheiter, 2010). To illustrate the utility of eye tracking in instructional design, Jarodzka et al. (2017) discuss the study by Jarodzka et al. (2015). In this study, it was investigated whether design principles based on CTMML could be applied to a computer-based test format. One such design principle is to avoid splitting information spatially across, e.g., a webpage. Students completed a computer-based exam with half of the questions presented in a split format (text and images spread left and right across the screen, respectively), and half in an integrated format (images placed with the text), while their gaze location was recorded with an eye tracker. The integrated format was expected to reduce visual search for information and lead to better performance. Student performance on the split and integrated formats was used to validate the design principle. The eye tracking data were used (1) to gain insights into visual search processes and (2) to estimate mental effort, and were used to test the reasoning behind the design principle.

Let’s consider the theories and linking hypotheses in more detail, beginning with CTMML. According to Mayer (2021, Table 5.3), CTMML makes three assumptions, namely (1) that humans possess separate channels for processing visual and auditory information (the dual channels assumption), (2) that humans are limited in the amount of information that can be processed in each channel at one time (the limited capacity assumption), and (3) that humans engage in active learning by attending to relevant information, organizing information, and integrating it with other knowledge (the active processing assumption). Based on these assumptions, the CTMML describes five processes that are necessary for multimedia learning: selecting words, selecting images, organizing words, organizing images, and integrating words and images. While selecting images may, intuitively, be related to gaze behavior and eye movements, the CTMML makes no explicit reference to eye movements or gaze. However, Jarodzka et al. (2015) illustrate that eye tracking may be used to test design principles based on CTMML:

“To investigate unnecessary visual search for related information, it is important to actually measure these processes, for example through eye tracking. Eye tracking reveals what a person looks at, for how long, and in which order (Holmqvist et al., 2011). As looking at certain elements is closely related to cognitively processing these elements, eye tracking captures visual and cognitive aspects of attention (Just & Carpenter, 1976)” (p. 805)

Thus, in addition to the CTMML, a linking hypothesis is needed to relate gaze behavior to the processes of selecting images or integrating words and images. Here, the generic version of Just and Carpenter’s original eye-mind hypothesis is assumed, according to which “the eye fixates the referent of the symbol currently being processed if the referent is in view” (Just & Carpenter, 1976, p.441). At the level of gaze measures, Jarodzka et al. (2015) operationalize the eye-mind hypothesis by assuming that transitions between the text and the picture are indicators of a large amount of visual search. While ‘visual search’ is not otherwise defined, they use it colloquially to describe looking around in the context of the learning material.

Contrary to the design principle derived from CTMML, students performed better for the exam questions in split format than in integrated format. Moreover, students did not make more transitions between text and images in the split format than in the integrated format questions. Thus, neither the effectivity of the design principle nor the hypothesized process behind this principle was corroborated by Jarodzka et al. (2015). What are the potential consequences of this failed corroboration for the CTMML or the linking eye-mind hypothesis? According to Jarodzka et al. (2015), one potential consequence is that “the assumption that a specific presentation format directly leads to a specific amount of visual search may be too simplistic” (p. 814). Additionally, several ad-hoc explanations are proposed that may be tackled in future research, “that should eventually result in a cognitive theory of multimedia testing, which in turn would deliver design guidelines for multimedia CBT [computer-based testing]” (p. 814).

In contrast to the previous examples, it is clear that the distance between scientific theory and eye-tracking data in instructional design is substantial. The theories do not directly predict anything about the eye movements or gaze behavior, nor are the linking hypotheses uncontested. This has the consequence that it is near impossible for a single study to falsify or corroborate the theory. Rather, it seems that the generic theory (CTMML) is undisputed and aspects of specific designs are evaluated using eye tracking. One assumes it may take many ‘failed’ designs to even begin to invalidate or discredit the theory (cf. Meehl, 1978, on how theories in ‘softer’ sciences tend to slowly fade away rather than be falsified).

It is important to note, however, that Jarodzka et al. (2015) are very explicit about the assumptions they make and how the eye-tracking data is used to gain insights into the use and effectivity of the learning material. In our opinion, this is often not the case in research fields where the link between theory and eye-tracking data is very indirect, or the chain of reasoning is long. Thus, the study by Jarodzka et al. (2015) allows us to make these points explicit.

## Implications

The goal of this article is for the reader to be able to answer the question whether they need or can use an eye tracker for their study, or at least what they should consider to answer that question. After considering the relation between scientific theory, the research question, and the use of eye-tracking technology in the previous examples, what are the implications for answering this question?

As stated at the outset, the question of whether to need an eye tracker or not may seem deceptively simple, while the answer may be difficult to produce. To us, asking this question and producing an answer is an iterative process that involves formulating a research question, consulting previous research, attempting to produce specific predictions, revisiting one’s research question, etc. We propose three different approaches for thinking about the relation between scientific theory, the research question, and the use of eye-tracking technology. First, we present a scheme to place one’s study in the context of other eye-tracking research. This represents a generic approach to the problem and may be useful for, e.g., determining what other literature to consult. Second, we present three considerations addressed in the examples above, which may aid one in conceiving their eye-movement study. Third and finally, we present a set of more specific questions that one may answer when conceiving their study. Importantly, we do not see the three different approaches as mutually exclusive, nor as there being a hierarchy. They can be seen as different strategies that may be more or less suitable for different readers.

### A generic scheme for eye-movement research

**Fig. 1:**
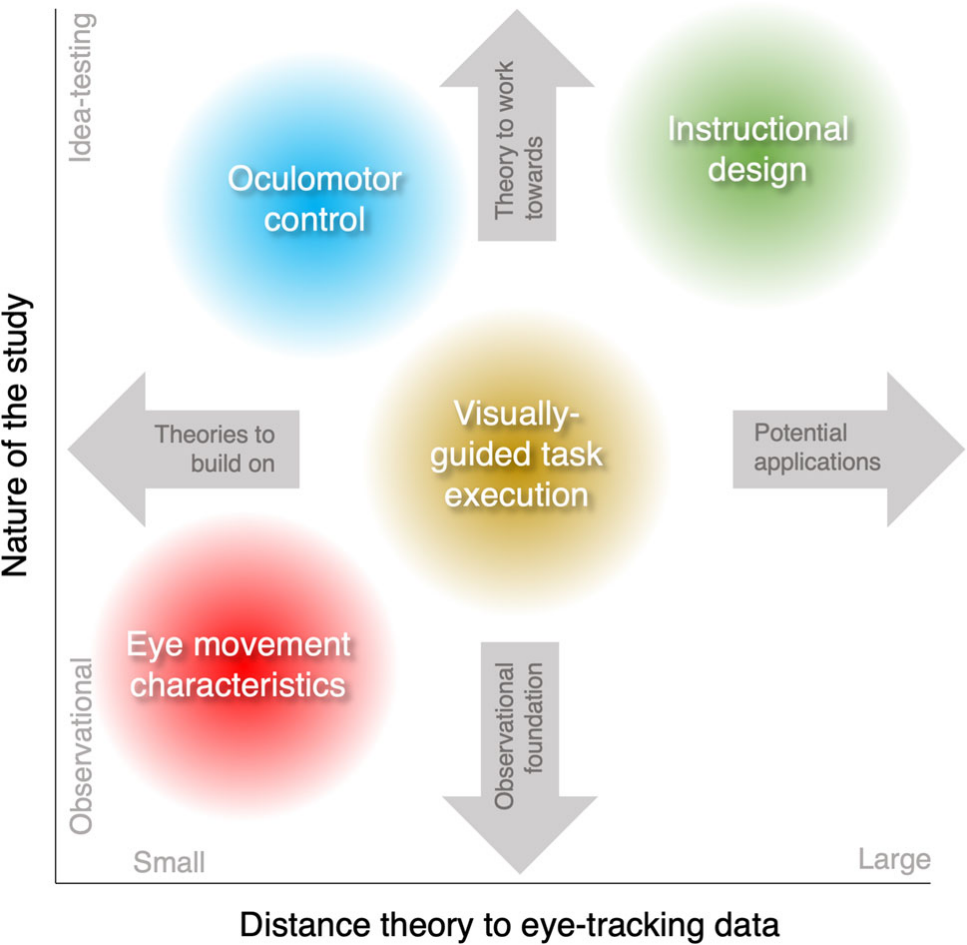
Schematic overview of example research topics from eye-movement research. The research topics are organized by the nature of the study (observational vs. idea-testing) and the distance between the theory and eye-tracking data

Consider Fig. 1, in which four of the example research topics are placed in a 2-D space, with the horizontal axis representing the distance between the research topic and the eye-tracking data and the vertical axis representing the nature of the study (observational vs. idea-testing) as a continuum. The examples are placed to illustrate their overall differences, not to draw conclusions about every study that has been conducted related to each topic. For example, studies on oculomotor control are more likely to make predictions close to the eye-tracking data (at the level of the signal or derived measures), while studies on instructional design are more likely to make predictions about, e.g., group-level statistics of the gaze behavior in various conditions of an experiment. In a similar vein, typical studies on visually guided task execution have a larger observational component than studies on oculomotor control. For simplicity, the examples from reading and scene perception are not depicted. For these research topics, it may depend on the perspective (e.g., oculomotor vs. cognitive) where one would place the research topic or study in this coordinate system.

Figure 1 represents one way of thinking about these various fields of eye-movement research. The current scheme is a generalization from a limited set of examples, and is certainly an oversimplification. First, it pertains only to research where theory plays an important role, or at least where it is desired. The scheme is meant to provide guidance for researchers starting out with eye tracking, and we believe it fulfills that requirement.

What implications could we derive on the basis of Fig. 1 for someone planning an eye-movement study? If one is able to determine roughly where one’s study is in this coordinate system, four implications can be derived, related to the four directions one can travel in this 2-D space (the four arrows in Fig. 1). First, in the leftward direction, one may find theories at smaller theory–data distances that one may be able to build and expand on. For example, for someone interested in journalism and marketing and how readers engage with various media platforms, the body of research conducted on reading may prove to be very useful (see part 2 in this article series (Hooge et al., 2024b), where this case is worked out in detail). Second, in the rightward direction, one may find possible applications of one’s observational or theoretical work, for example, the journalism or marketing field for someone investigating eye movements during reading. Third, in the downward direction, one may find the observational foundation for a particular study. If that observational foundation does not exist, perhaps it is worth considering beginning with such a study. Fourth, in the upward direction, one may find overarching theoretical frameworks that can deliver predictions for an eye-movement study. If they are absent, such a theoretical framework is worth striving towards. One example of a research topic where this bidirectional relation between observational and theoretical work is observed is on idiosyncrasy in face-scanning behavior. Walker-Smith et al. (1977) observed large differences in face-scanning behavior for their three participants. This has later been shown to generalize from static stimuli to encounters in the world (i.e., observational work conducted by Peterson et al., 2016), and is being integrated into theoretical work on social-information seeking and biological niche construction (e.g., Constantino et al., 2017; Hessels et al., 2020a). Understanding the four directions in Fig. 1 relative to one’s study may help to position the work better.

The scientific literature may be a good place to start searching for specific examples pertaining to the four described directions relative to one’s study. If it turns out the distance to other research is large – whether to research of a more observational character than the planned study (downward), or to other research topics one may build upon (leftward) – one might consider changing or simplifying the study. More specifically, for someone new to eye-tracking, it may be sensible to position oneself close to other research. In that way, there is either some directly comparable finding, or an extrapolation of other people’s findings to one’s research. If one cannot place the study in the present framework at all, perhaps the use of an eye tracker needs to be reconsidered. Note that this does not mean that every study with an eye tracker ought to be placeable in this framework. For example, there may be engineering questions for which theory plays no role or a very minor one, but which certainly require an eye tracker, such as the development and validation of gaze-based interaction systems (e.g., Stampe & Reingold, 1995; Ohno et al., 2002; Ohno & Mukawa, 2004). Similarly, eye trackers may be used solely to ensure that participants maintain fixation on a specified location, such as a fixation cross (e.g., Patching & Jordan, 1998; Christ & Abrams, 2006; Galfano et al., 2012).

### Three considerations for conceiving an eye-movement study

The examples outlined in this article also allow us to derive three considerations. First, it should be clear whether eye tracking is used to observe and describe eye movements, or whether eye movements or gaze behavior are used as a proxy for something else, e.g., oculomotor, perceptual or cognitive control. This distinction is not trivial. Using eye movements or gaze behavior as a proxy for something else may require either explicit models of the link between the eye movements and the higher control system (such as for oculomotor control), or a form of eye-mind hypothesis to bridge the gap.

Although, at a surface level, both the immediacy assumption and the eye-mind assumption (Just & Carpenter, 1980) may appear to be reasonable approximations, research from many different fields has accumulated significant evidence against strong versions of these hypotheses (e.g., Viviani, 1990; Underwood & Everatt, 1992; Fox et al., 1996; Hooge & Erkelens, 1996; Anderson et al., 2004; Irwin, 2004; Reichle & Reingold, 2013; Schindler & Lilienthal, 2019; Wu & Liu, 2022). In any case, when adopting a form of eye-mind hypothesis, it ought to be made clear to the reader. We often find that assumptions about e.g., the link between gaze behavior and cognition are implicit or not evident (cf. Aslin, 2007; Griffin, 2004). Thus, we encourage researchers to make assumptions explicit, and to investigate whether more established theories (such as on the link between eye movements and perceptual processing) may be applicable to one’s research. The latter avoids having to reinvent the wheel when eye tracking as a technique is adopted into a new research field.

Second, it is important, but not trivial, to understand the chain of reasoning (or link) between scientific theory and eye-tracking data. The examples made clear that theories may make predictions at the level of the eye-tracker signal (see section on Oculomotor control), at the level of measures that can be derived from the eye-tracking signal (see sections on Oculomotor control, Reading, and Expertise), or at the level of the statistics of overall gaze behavior (see section on Task-related gaze behavior). In some cases, the chain of reasoning may not allow one to link the main theory to the eye-tracking data at all (see section on Instructional design), but additional or ad-hoc hypotheses are needed. Again, we encourage the researcher to make the chain of reasoning explicit or consult existing theoretical frameworks in more established research fields when motivating one’s predictions or research questions. The distance between theory and eye-tracking data may also be characterized as whether certain concepts can be operationalized directly or indirectly, a point that will be addressed in more detail in part 2 in this article series (Hooge et al., 2024b).

A third consideration is whether an eye-movement study is in fact of a theory- or hypothesis-testing nature, or of a more exploratory nature. Note that a hypothesis may be as simple as a proposed explanation for an observed phenomenon (as in the eye-witness example on Scene Perception). It does not necessarily have to be as broad of a theory as e.g., the Cognitive Theory of Multimedia Learning (example on Instructional Design), or require computational modeling (as in the example on Reading). As outlined before, the distinction between theory-testing and exploratory research may be controversial (see e.g., Platt, 1964; Meehl, 1978; Felin et al., 2021; Hessels & Hooge, 2021), and in our experience, exploratory research is often seen as less scientific then theory-testing research (unduly, in our opinion). Moreover, it also seems that in research fields with less of a history of eye tracking, or generally longer or weaker chains of reasoning from theory to eye-tracking data, a lot of emphasis is in fact placed on the theory (see e.g., Kok & Jarodzka, 2017; Godfroid & Hui, 2020). In other words, it seems that studies with a large distance between theory and eye-tracking data of an observational nature are lacking (the bottom right quadrant in Fig. 1). Kok and Jarodzka (2017) express this position explicitly, writing about eye tracking in the context of medical education: “In order to interpret eye-tracking data properly, theoretical models *must always* be the basis for designing experiments as well as for analyzing and interpreting eye-tracking data” (p. 114, emphasis ours). We disagree with this position, as there is not always a theory that makes clear predictions at the level of the eye-tracking data (as we believe is the case for the CTMML in instructional design). We would advise researchers for whom this holds to consider a more exploratory approach in their work. This can be tremendously valuable, as evidenced by some of the early studies on eye movements (Huey, 1900; Dodge, 1903) as well as the studies by Land et al. (1999) and Pelz and Canosa (2001) for research on visually guided task execution. Exploration can lay the necessary foundation for new theories to emerge, both in research fields where eye tracking has been applied for a long time, as well as research fields where eye tracking may be new. For newer research fields, it may be that a lot of the relevant groundwork has not yet been done. It may be that by starting from research with a more observational character, better and more explicit theories can be formulated, pushing one from the bottom to the top of Fig. 1. Finally, there are at least two situations in which we would urge the researcher to reconsider the use of an eye tracker: (1) if the link between theory and eye-tracking data is not clear, and (2) if there is no theory, and exploration and description are not of scientific interest.

### A set of questions for conceiving an eye-movement study

Positioning one’s study in the 2-D space depicted in Fig. 1 may be one useful tool to achieve the goal of answering the question of whether one needs or can use an eye tracker for their study. However, if one finds this difficult to put into practice, one may also first consider the more specific questions below to help formulate and explicate their research questions.

For researchers using eye tracking in theoretically driven research, the following questions may be worthwhile to consider. The examples given above may serve as useful reading material for answering these questions.

1. Is there a link between the theory and eye-movement or gaze measures, and if so, what is it? If not, then using an eye tracker is likely not informative about the theory.
2. At what level does the theory make predictions? At the level of the eye-tracker signal, an eye-movement measure, aggregates of gaze behavior, group differences, or a statistical relation with another measure?
3. Are only eye-tracking data necessary and/or required to answer the research question?
4. Are additional assumptions or auxiliary hypotheses needed? If so, what are these?
5. Are the assumptions made common in one’s research field? Are they common in more established research fields? If so, have they been supported or discredited?

For researchers considering using eye tracking in exploratory research, the following questions may be worthwhile to consider. When applicable, we give examples that may serve as inspiration.

1. Can one perform the task or activity without making eye movements? Would performance or other aspects of behavior be impeded if one does not move the eyes? If not, it may be that an eye tracker does not yield informative insights into the problem. However, even if one does not necessarily need to move one’s eyes, biases in gaze behavior may occur (see e.g., Hessels, 2020, for an elaborate example in the context of gaze to faces).
2. What aspects of eye movements or patterns of gaze behavior (e.g., biases) may be informative to describe? Have such patterns been described before? Part 2 in this article series goes into more detail on these kinds of questions (Hooge et al., 2024b).
3. Do such patterns of gaze behavior change as a function of certain factors of interest (e.g., a viewing bias as a function of age, or a pattern of gaze behavior as a function of country of upbringing or expertise)? See, for example, Van Renswoude et al. (2016), who investigated the horizontal saccade bias in infants compared to adults during scene perception, or Allsop and Gray (2014) who compared the order (more specifically, entropy) of gaze patterns in a simulated airplane cockpit in anxiety-induced versus control conditions.
4. Are the patterns of gaze behavior potentially correlated to other patterns of behaviors of interest (e.g., social behavior) or to other outcome measures (e.g., a gold standard in a particular research field)? See, for example, Maran et al. (2022), who correlated population density of the town/city people grow up in with how likely they were to fixate on the faces of others, or Klin et al. (2002), who correlated looking time to the mouth area to a clinical gold standard (Autism Diagnostic Observation Schedule scores).
5. Is there a norm group against which a sub-group, or certain individuals can be compared? See, for example, Tant et al. (2002), who compared scanning behavior during a dot-counting task of healthy controls against a group of homonymous hemianopia (HH) patients. In addition, HH was simulated for the control group and scanning behavior was compared against their own ‘normal’ and the HH patients’ behavior.
6. If there are no expectations for what patterns might emerge, are there (a)symmetries or anisotropies to consider? See, for example, Hessels et al. (2016) who investigated (among others) the symmetry in amplitude and direction change of infant’s eye movements during spontaneous search.

## Concluding remarks

In this article, we have illustrated the link between scientific theory, research question, and predictions for a varied selection of eye-movement studies. These examples allowed us to emphasize three aspects on an eye-movement study that are relevant to consider. First, is the goal the description of eye-movements or gaze behavior, or are eye-movements and gaze behavior used as proxy for e.g., aspects of cognition? Second, what is the chain of reasoning from scientific theory to predictions about eye-movement or gaze measures? Third, is the study of an observational or idea-testing nature? In addition, the elaborate examples from oculomotor control, reading, scene perception, visually guided task execution, expertise, and instructional design may serve as inspiration to researchers new to eye-tracking. Finally, to fully appreciate the points made in this article, we suggest that the reader consult the article again after having started preparing an eye-movement study. For example, the reader may read part 2 of this article series (Hooge et al., 2024b), start setting up a study, and revisit the present article. To us, understanding the relation between scientific theory, research question, and eye-movement and gaze measures is an iterative process.

## Data Availability

Not applicable.

## Footnotes

1 More elaborate histories of eye-tracking methodology can be found in e.g., Wade and Tatler (2005), Wade (2007), and Holmqvist et al. (2011).

2 A.D. de Groot’s dissertation ‘Het denken van de schaker’ (original Dutch title) was published in 1946. The work was translated to English in 1965 and has since appeared in various prints.

3 The labels ‘theory’, ‘hypothesis’, and ‘model’ are copied verbatim from the article by Gegenfurtner et al. (2011). What the differences between a theory, hypothesis, and model are, is beyond the scope of the present article (but see, e.g., Bolles, 1962; Reese & Overton, 1970; Meehl, 1978).

## Declarations

### Open practices statement

There are no data or code associated with the present article.
